# Identification of fertility restoration candidate genes from a restorer line R186 for *Gossypium harknessii* cytoplasmic male sterile cotton

**DOI:** 10.1186/s12870-023-04185-z

**Published:** 2023-04-04

**Authors:** Cheng Cheng, Hushuai Nie, Huijing Li, Daniel Adjibolosoo, Bin Li, Kaiyun Jiang, Yanan Cui, Meng Zhu, Baixue Zhou, Anhui Guo, Jinping Hua

**Affiliations:** Laboratory of Cotton Genetics, Genomics and Breeding /Key Laboratory of Crop Heterosis and Utilization of Ministry of Education, College of Agronomy and Biotechnology, China Agricultural University, Haidian District, No. 2, Yuanmingyuan West Rd, Beijing, 100193 China

**Keywords:** *Gossypium harknessii*, CMS, BSA, *Rf candidate genes*, RNA-seq

## Abstract

**Background:**

The utilization of heterosis based on three-line system is an effective strategy in crop breeding. However, cloning and mechanism elucidation of restorer genes for cytoplasmic male sterility (CMS) in upland cotton have yet been realized.

**Results:**

This research is based on CMS line 2074A with the cytoplasm from *Gossypium harknessii* (D_2-2_) and restorer line R186. The offspring of 2074A × R186 were used to conduct genetic analysis. The fertility mechanism of 2074A can be speculated to be governed by multiple genes, since neither the single gene model nor the double genes model could be used. The bulked segregant analysis (BSA) for (2074A × R186) F_2_ determined the genetic interval of restorer genes on a region of 4.30 Mb on chromosome D05 that contains 77 annotated genes. Four genes were identified as candidates for fertility restoration using the RNA-seq data of 2074A, 2074B, and R186. There are a number of large effect variants in the four genes between 2074A and R186 that could cause amino acid changes. Evolutionary analysis and identity analysis revealed that GH_D05G3183, GH_D05G3384, and GH_D05G3490 have high identity with their homologs in D_2-2_, respectively. Tissue differential expression analysis revealed that the genes *GH_D05G3183*, *GH_D05G3384,* and *GH_D05G3490* were highly expressed in the buds of the line R186. The predicted results demonstrated that GH_D05G3183, GH_D05G3384 and GH_D05G3490 might interact with GH_A02G1295 to regulate *orf610a* in mitochondria.

**Conclusion:**

Our study uncovered candidate genes for fertility restoration in the restorer line R186 and predicted the possible mechanism for restoring the male fertility in 2074A. This research provided valuable insight into the nucleoplasmic interactions.

**Supplementary Information:**

The online version contains supplementary material available at 10.1186/s12870-023-04185-z.

## Background

Plant male sterility mainly includes three types: cytoplasmic male sterility (CMS), cytoplasmic nuclear interaction male sterility, and nuclear male sterility (GMS) [1]. CMS is the most convenient method to produce the population of male sterility in the commercial application of hybrids [2]. CMS gene is reported to be associated with chimeric mitochondrial *orf* [3]. Sterility of plants with CMS cytoplasm results from the influence of CMS proteins on mitochondria such as toxic effect [4, 5], burst of reactive oxygen species (ROS) [6], inducing the abnormal programmed cell death (PCD) [7] and retrograde regulating nuclear genes [8]. CMS-A_2_ [9], CMS-B_1_ [9], CMS-D_2-2_ [10], CMS-D_8_ [11] and CMS-AD_1_ [12] constitute main types of cotton CMS. CMS-D_2-2_ and CMS-D_8_ are the most valuable for production and are popular in cotton three-line hybrid breeding among many CMS types of cotton. The cytoplasm of *Gossypium harknessii* Brandegee (D_2-2_) was transferred into AD_1_ nuclear background, which led to the production of CMS-D_2-2_ [10]. Li [13, 14] constructed fosmid library of mitochondrial genome from D_2-2_ male sterile line 2074A and identified 28 ORFs specific to CMS 2074A by mitochondrial genome analysis.

*Restorer of fertility* (*Rf*) genes rescue the fertility of CMS cytoplasm plants by interacting with CMS gene products and alleviating or eliminating the adverse effects on mitochondria of CMS gene products at DNA [15], transcription [8], post-transcription [16], translation [17–19], post-translation [20], and metabolic [21] levels. The mechanisms of fertility restoration are diverse and the types of *Rf* genes are also not fixed. More than half of cloned restorer genes encode pentatricopeptide repeat (PPR) proteins [22]. There are also some reports that aldehyde dehydrogenase [21], acyl-carrier protein synthase (ACPS)-like domain containing protein [23], glycine-rich protein [24], peptidase-like protein [20], bHLH transcription factor [25] and transcription factors of the plant DREB1 family [8] are designated as *Rf* genes and can also restore the fertility of plants with CMS genes.

Although some reports found that multiple genes can rescue sterility of CMS-D_2-2_ cotton [26], Weaver [27] and Zhang [28] confirmed fertility of cotton plant with CMS-D_2-2_ cytoplasm is conditioned by a single dominance locus at sporophytical level. The gene located at the single dominant locus is designated as *Rf1*. CMS-D_8_ was bred by combining cytoplasm of *Gossypium trilobum* (DC) Skovst (D_8_) and AD_1_ nucleus [11]. A single dominant locus containing *Rf2* gene can gametophytically dominate fertility of cotton plant with CMS-D_8_ cytoplasm [29]. The sterility of CMS-D_8_ cotton can be remedied by *Rf1* and *Rf2* while fertility restoration of CMS-D_2-2_ cotton only depends on the function of the *Rf1* gene [30].

The tightly linked *Rf1* and *Rf2* are located in the 0.93 cM interval of same chromosome [28]. Each of the *Rf* genes is not linked to any morphological markers with known chromosomal locations in cotton [31]. A variety of molecular markers are used for genetic mapping of *Rf* genes in cotton, such as RAPD [32–34], SSR [32, 34], STS [34, 35], AFLP [36], SNP [37, 38] and InDel [39, 40]. Liu [32] mapped the genetic interval of *Rf1* gene on the long arm of chromosome 4 (A subgenome). The mapping interval of *Rf1* locus was delimited to 100 kb and was between 081-05 K and 052-01N BAC clones [34]. Wang [30] found that *Rf1* and *Rf2* were delimited to a 1.4 cM genetic distance on chromosome D05 with assistant of four SSR markers. The locus of *Rf* gene was located on 1.35 Mb of chrD05 by the technology of BSA with SLAF-seq [37]. Feng [38] determined the location of *Rf2* on a 1.48 Mb interval of chromosome D05, based on BSA with high-throughput SNP genotyping. While much efforts has been devoted to the genetic mapping of *Rf* loci, valuable *Rf* candidates have rarely been identified in cotton. Upgrading of sequencing technology, reduction of costs, and continuous release of high-quality cotton reference genomes [41–44] provide better opportunities for the localization and cloning of cotton *Rf* genes.

In this research, the key *Rf* candidates that dominate the fertility of CMS-D_2-2_ cotton were mapped by BSA and analyzed for genetics, expression, sequence similarity, and evolution. First, strong restorer lines were screened with a fertility survey of F_1_ hybrids produced by crossing the CMS-D_2-2_ line with the restorer line. Genetic analysis of the F_2_ and BC_1_F_1_ populations originating from the CMS-D_2-2_ line and the strong restorer lines proved that the fertility of CMS-D_2-2_ cotton is controlled neither by a single locus nor by two loci. Then, the genetic interval of the *Rf* genes was determined using the BSA technology. Next, RNA-seq and sequence variation information supported the identification of four *Rf* candidate genes. The homologous proteins of four candidate proteins in *Gossypium herbaceum* (A_1_) [42], *Gossypium arboreum* (A_2_) [42], *G. hirsutum* (AD_1_) [43] and all D genome cotton [45–47] were used for evolutionary and sequence similarity analysis. *GH_D05G3183*, *GH_D05G3384*, and *GH_D05G3490* were identified as candidate fertility restorer genes by evolutionary and sequence similarity analysis. Real-time quantitative PCR (qRT-PCR) proved that *GH_D05G3183*, *GH_D05G3384*, and *GH_D05G3490* genes were highly expressed in R186 buds. Protein interaction analysis revealed that GH_D05G3183, GH_D05G3384, and GH_D05G3490 may interact with GH_A02G1295 to regulate orf160a and thus restore male fertility in cotton. From the above evidence, we can conclude that *GH_D05G3183*, *GH_D05G3384*, and *GH_D05G3490* genes are likely *Rf* genes of CMS-D_2-2_. This research lays the foundation for the subsequent identification of restoration genes, the elucidation of restoration mechanisms, and the breeding of strong restorer lines with excellent agronomic traits.

## Results

### Genetic analysis of *Rf* loci

*Rf* loci can rescue the fertility of 2074A with CMS-D_2-2_ cytoplasm sporophytically [28]. The fertility surveys of (2074A × R186) F_2_ in three environments and the BC_1_F_1_ population (2074A × (2074A × R186) in two environments were conducted (Additional file 3, Supplemental Table 1). First, we assumed that the *Rf* locus is single-gene dominant inheritance. The chi-square test results of F_2_ and BC_1_F_1_ population derived from R186 could not unanimously prove this hypothesis (Table 1). Then, we assumed that the fertility of 2074A was controlled by two dominant loci. The ratios of F_2_ genotypes of double genes interaction including no interaction, dominant complementary effect, inhibiting effect, epistatic recessiveness, epistatic dominance, duplicate effect and additive effect are 9:3:3:1, 9:7, 13:3, 9:3:4, 12:3:1, 15:1 and 9:6:1, respectively. Because the fertility data distribution trend of two F_2_ population is more similar to the model of dominant complementary effect, epistatic recessiveness and duplicate effect, the fertility survey results of two F_2_ and two BC_1_F_1_ population were tested by chi-square, based on these double genes interaction model (Additional file 3, Supplemental Tables 2–4). However, the genetic model of *Rf* genes from R186 failed to fit any model of dominant complementary effect, epistatic recessiveness, and duplicate effect. The analytical results of this research proved that the sterility in 2074A with CMS-D_2-2_ cytoplasm can be remedied by multiple genes, neither single nor double.

**Table 1:** Genetic analysis of *Rf* genes from R186 based on single gene dominant inheritance hypothesis

Population	Phenotype	O	E	O-E	(O-E)^2^/E	(|O-E|-0.5)^2^/E	χ^2^
F_2_ (2020 Sanya)	Fertile	365	299	66	14.57	14.35	57.25
	Sterile	34	100	-66	43.56	42.9	
BCF_1_ (2020 Sanya)	Fertile	320	243	77	24.4	24.08	47.54
	Sterile	167	243	-76	23.77	23.46	
F_2_ (2020 Hejian)	Fertile	341	343	-2	0.01	0.01	0.02
	Sterile	116	114	2	0.04	0.01	
BCF_1_ (2020 Hejian)	Fertile	212	194	18	1.67	1.58	3.16
	Sterile	176	194	-18	1.67	1.58	
F_2_ (2021 Hejian)	Fertile	244	220	24	2.62	2.51	9.97
	Sterile	50	74	24	7.78	7.46	

Notes: O, observed value. E, expected value. df = 1, *P* = 0.05, χ2 = 3.84. df = 1, *P* = 0.01, χ2 = 6.64

**Table 2:** Genome-wide distribution of SNPs and InDels

Chr	Length (bp)	(2074A × R186) F_2_			
		**No. SNPs**	**SNP density**	**No. InDels**	**InDel density**
A01	118,174,371	2729	23.09	497	4.21
A02	108,272,889	1719	15.88	334	3.08
A03	111,586,618	26,462	237.14	3764	33.73
A04	87,703,368	1954	22.28	407	4.64
A05	110,845,161	114,496	1032.94	9734	87.82
A06	126,488,190	6497	51.36	1084	8.57
A07	96,598,283	14,246	147.48	1864	19.30
A08	125,056,055	149,600	1196.26	11,509	92.03
A09	83,216,487	7631	91.70	1141	13.71
A10	115,096,118	10,498	91.21	1543	13.41
A11	121,376,521	6658	54.85	1189	9.80
A12	107,588,319	8454	78.58	1374	12.77
A13	110,367,549	11,712	106.12	1546	14.01
D01	64,698,102	3622	55.98	622	9.61
D02	69,777,850	17,836	255.61	2560	36.69
D03	53,896,199	8845	164.11	1720	31.91
D04	56,935,404	3483	61.17	636	11.17
D05	63,929,679	318,616	4983.85	25,800	403.57
D06	65,459,843	12,059	184.22	2028	30.98
D07	58,417,686	18,442	315.69	2523	43.19
D08	69,080,421	26,922	389.72	3213	46.51
D09	52,000,373	8767	168.59	1395	26.83
D10	66,881,427	9471	141.61	1459	21.81
D11	71,358,197	3746	52.50	787	11.03
D12	61,693,100	9190	148.96	1630	26.42
D13	64,447,585	1746	27.09	358	5.55
Whole	2,240,945,795	805,401	359.40	80,717	36.02

### Evaluation of whole genome resequencing data and BSA mapping of *Rf* genes

**Fig. 1:**
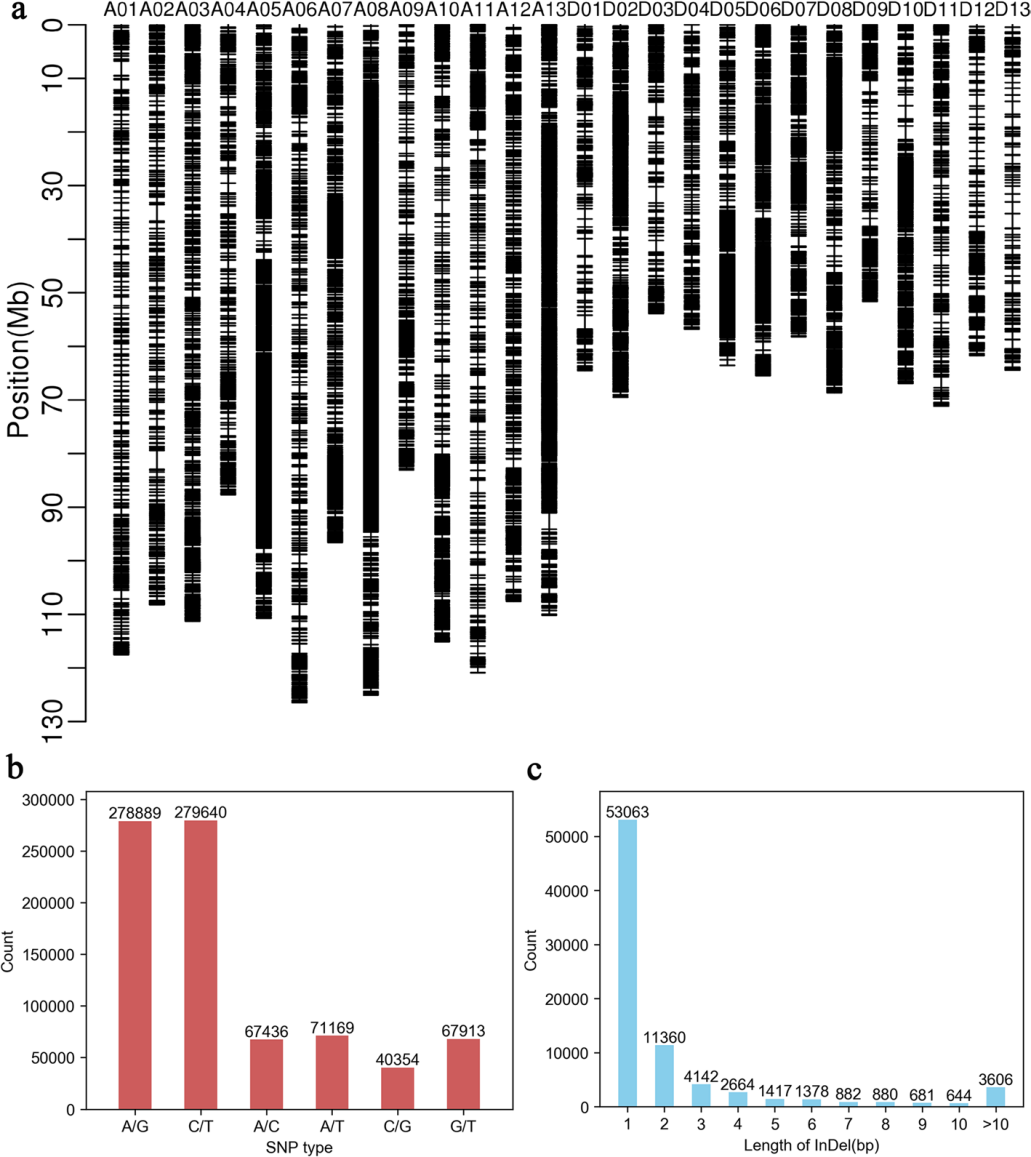
The SNP and InDel distribution and types of the (2074A × R186) F_2_ segregating population. **a** Distribution of the SNP and InDel for the F_2_ segregation population. **b** Statistics of SNP types for the F_2_ segregation population. **c** Statistics of InDel types for the F_2_ segregation population

Two parent lines 2074A and R186, together with extremely fertile and extremely sterile bulks of (2074A × R186) F_2_ were sequenced on the Illumina HiSeq platform for BSA. A total of 850,956,496 reads and 252.6 Gb data were obtained (Additional file 4, Supplemental Table 5). The average GC content, Q30, genome coverage and coverage depth were 36.10%, 93.16%, 94.38% and 24.47 × in the sequencing results, respectively. A total of 173,351,714 reads and 51.40 Gb data were generated for 2074A with average GC content of 36.21%, a Q30 value of 92.70%, genome coverage of 94.86% and coverage depth of 20.13 × (Additional file 4, Supplemental Table 5). The sequencing results showed that R186 possessed 174,143,920 reads, average GC content of 36.32%, Q30 of 93.01%, coverage depth of 20.34 × and genome coverage of 93.62% (Additional file 4, Supplemental Table 5). On the other hand, 240,953,554 and 262,507,308 reads were gained for extremely fertile and extremely sterile bulks of (2074A × R186) F_2_, respectively, with Q30 values of 93.62% and 93.31%, average GC content of 35.62% and 36.26%, coverage depth of 27.51 × and 29.91 × , and genome coverage of 94.07% and 94.95% (Additional file 4, Supplemental Table 5). Those reads were aligned to the reference AD_1_ genome [43]. A total of 805,401 SNPs and 80,717 InDels were obtained from the two bulks of (2074A × R186) F_2_ (Additional file 4, Supplemental Table 6). D05 chromosome has the highest number and density of SNPs and InDels among the variants detected in two bulks from (2074A × R186) F_2_ (Fig. 1a, Table 2). Both A/G and C/T type SNPs accounted for the highest proportions among the SNPs detected in two bulks from (2074A × R186) F_2_ (Fig. 1b). The InDels with length of 1 bp accounted for the largest proportion among the InDels detected in two bulks (Fig. 1c).

**Fig. 2:**
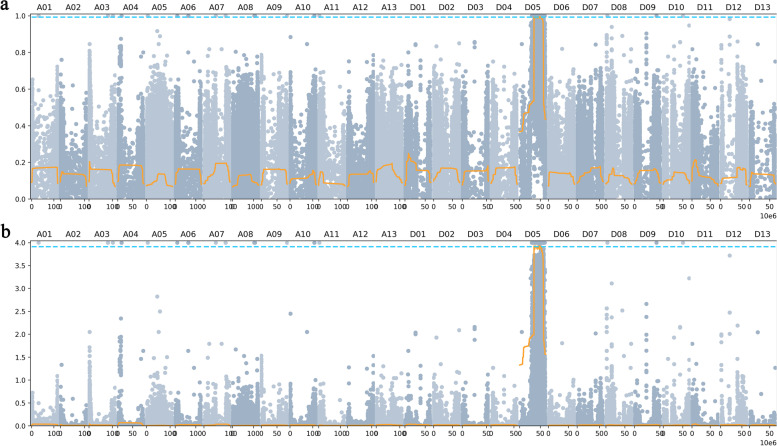
The location determination of *Rf* genes from the R186 by ΔSNP-index and ED algorithms. **a** The mapping of *Rf* genes using ΔSNP-index algorithm. **b** The mapping of *Rf* genes using ED algorithm

The algorithms of Δ(SNP-index) and euclidean distance (ED) algorithms were used to locate intervals of *Rf* genes in BSA. The *Rf* genes from R186 were delimited on 34,943,848–35,280,626 bp, 37,694,536–38,093,258 bp, 38,227,690–38,918,070 bp, 43,648,410–43,742,747 bp, 44,220,658–44,835,843 bp, 45,653,643–45,811,858 bp, 46,818,876–47,801,436 bp, 49,315,266–50,052,334 bp, 51,046,570–51,244,308 bp and 51,557,591–51,715,302 bp of chromosome D05 using Δ(SNP-index) algorithm (Fig. 2a, Additional file 5, Supplemental Table 7). ED algorithm determined the position of *Rf* genes on 34,937,629–35,327,222 bp, 37,656,846–38,088,236 bp, 38,266,076–38,923,764 bp, 43,619,667–43,777,276 bp, 44,162,548–44,834,728 bp, 45,654,955–45,821,873 bp, 46,834,875–47,798,159 bp, 49,311,406–50,065,365 bp, 51,034,546–51,244,603 bp and 51,563,873–51,719,761 bp of chromosome D05 (Fig. 2b, Additional file 5, Supplemental Table 8). Eventually, the *Rf* genes from R186 were mapped in the 4.30 Mb interval of chromosome D05, based on the Δ(SNP-index) and ED algorithms, including intervals 34,943,848–35,280,626 bp, 37,694,536–38,088,236 bp, 38,266,076–38,918,070 bp, 43,648,410–43,742,747 bp, 44,220,658–44,834,728 bp, 45,654,955–45,811,858 bp, 46,834,875–47,798,159 bp, 49,315,266–50,052,334 bp, 51,046,570–51,244,308 bp and 51,563,873–51,715,302 bp on chromosome D05, which contain a total of 77 genes (Table 3).

**Table 3:** The location determination of Rf genes from the R186 by BSA

QTL number	Chr	Left (bp)	Right (bp)	Physical interval (bp)	Gene number	Gene ID
1	D05	34,943,848	35,280,626	336,778	10	*GH_D05G3174; GH_D05G3175; GH_D05G3176; GH_D05G3177; GH_D05G3178; GH_D05G3179; GH_D05G3180; GH_D05G3181; GH_D05G3182; GH_D05G3183*
2	D05	37,694,536	38,088,236	393,700	2	*GH_D05G3255; GH_D05G3256*
3	D05	38,266,076	38,918,070	651,994	12	*GH_D05G3262; GH_D05G3263; GH_D05G3264; GH_D05G3265; GH_D05G3266; GH_D05G3267; GH_D05G3268; GH_D05G3269; GH_D05G3270; GH_D05G3271; GH_D05G3272; GH_D05G3273*
4	D05	43,648,410	43,742,747	94,337	1	*GH_D05G3310*
5	D05	44,220,658	44,834,728	614,070	14	*GH_D05G3319; GH_D05G3320; GH_D05G3321; GH_D05G3322; GH_D05G3323; GH_D05G3324; GH_D05G3325; GH_D05G3326; GH_D05G3327; GH_D05G3328; GH_D05G3329; GH_D05G3330; GH_D05G3331; GH_D05G3332*
6	D05	45,654,955	45,811,858	156,903	5	*GH_D05G3350; GH_D05G3351; GH_D05G3352; GH_D05G3353; GH_D05G3354*
7	D05	46,834,875	47,798,159	963,284	12	*GH_D05G3378; GH_D05G3379; GH_D05G3380; GH_D05G3381; GH_D05G3382; GH_D05G3383; GH_D05G3384; GH_D05G3385; GH_D05G3386; GH_D05G3387; GH_D05G3388; GH_D05G3389*
8	D05	49,315,266	50,052,334	737,068	14	*GH_D05G3456; GH_D05G3457; GH_D05G3458; GH_D05G3459; GH_D05G3460; GH_D05G3461; GH_D05G3462; GH_D05G3463; GH_D05G3464; GH_D05G3465; GH_D05G3466; GH_D05G3467; GH_D05G3468; GH_D05G3469*
9	D05	51,046,570	51,244,308	197,738	4	*GH_D05G3488; GH_D05G3489; GH_D05G3490; GH_D05G3491*
10	D05	51,563,873	51,715,302	151,429	3	*GH_D05G3504; GH_D05G3505; GH_D05G3506*

### Go annotation of genes in the association interval

**Fig. 3:**
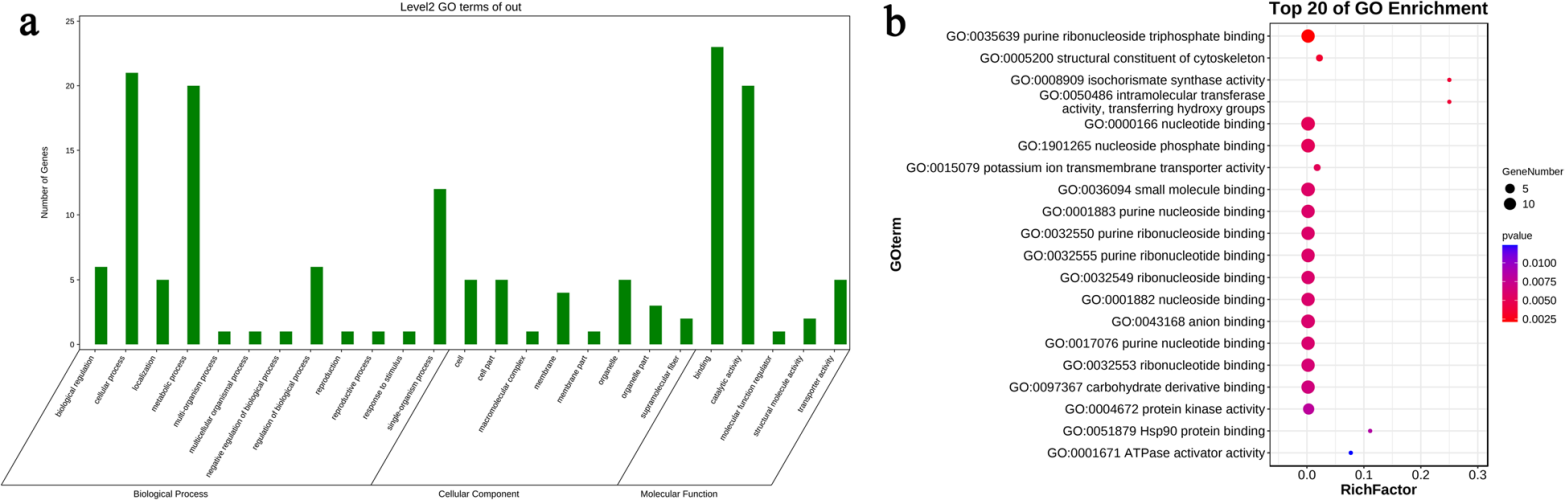
GO annotation results for genes in candidate regions. **a** Gene number in each category of GO annotations. **b** GO annotation in the category of molecular function

Fourty-two genes from seventy-seven genes located in BSA interval were identified through gene ontology (GO) analysis (Fig. 3a). Twenty-eight, thirty-seven, and nine genes, respectively, are involved in biological process, molecular function, and cellular component. The significantly enriched molecular function mainly includes binding, molecular function regulator, catalytic activity, structural molecule activity and transporter activity (Fig. 3a). The purine ribonucleoside triphosphate binding genes were speculated to be involved in male fertility, which includes *GH_D05G3176*, *GH_D05G3177*, *GH_D05G3263*, *GH_D05G3269*, *GH_D05G3270*, *GH_D05G3273*, *GH_D05G3328*, *GH_D05G3386*, *GH_D05G3461*, *GH_D05G3467*, *GH_D05G3468*, *GH_D05G3469* and *GH_D05G3491* genes (Fig. 3b). The Rf proteins, which regulate male fertility at the post-transcriptional level, usually modulate the stability of the abortive gene mRNA by binding to it. The purine ribonucleoside binding and ribonucleoside binding are molecular functions that need to be focused, including *GH_D05G3176*, *GH_D05G3177*, *GH_D05G3263*, *GH_D05G3269*, *GH_D05G3270*, *GH_D05G3273*, *GH_D05G3328*, *GH_D05G3386*, *GH_D05G3461*, *GH_D05G3467*, *GH_D05G3468*, *GH_D05G3469* and *GH_D05G3491* (Fig. 3b). In addition, the relationship between other types of molecular function and male fertility needs to be clarified in follow-up studies.

### Determination of *Rf* candidate genes by expression analysis

More than half of the reported fertility restoration genes for cytoplasmic male sterility belong to the PPR genes. Only one PPR gene, *GH_D05G3465* gene, was identified in the 4.30 Mb interval, based on the reference AD_1_ genome [43]. However, the identity between GH_D05G3465 and homologous protein in D_2-2_ was only 22.96% (Additional file 6, Supplemental Table 9). No significant difference in *GH_D05G3465* gene expression levels was detected in buds of 2074A, 2074B and R186 (Additional file 6, Supplemental Table 9). Therefore, *GH_D05G3465* was hardly considered as *Rf* candidate gene.

**Fig. 4:**
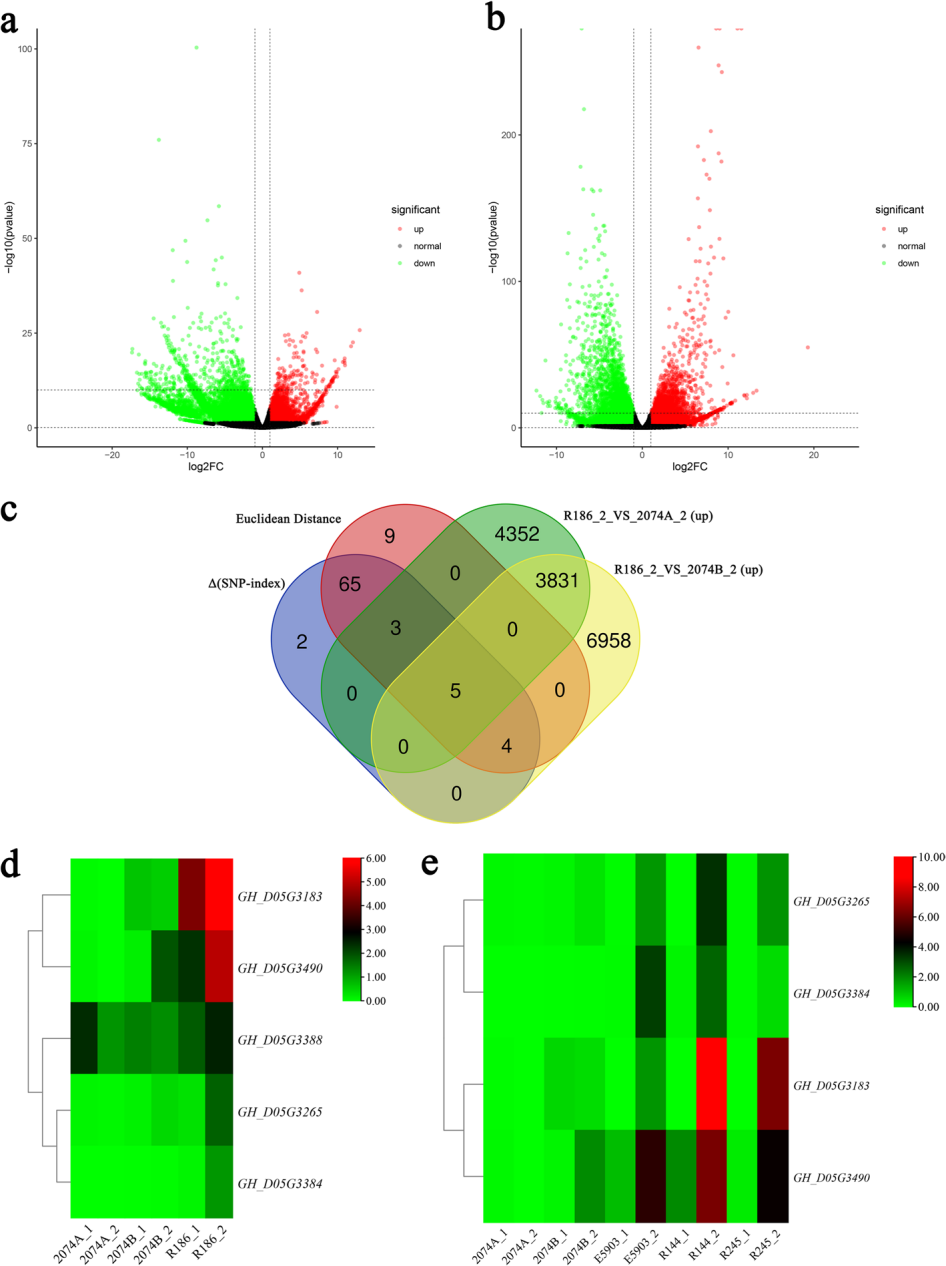
Identification of CMS-Rf candidate genes based on RNA-seq. **a** Volcanic map of R186_2 vs. 2074A_2. **b** Volcanic map of R186_2 vs. 2074B_2. **c** Determination of *Rf* candidate genes in BSA interval by venn diagram, based on gene expression in 2074A, 2074B and R186. **d** The heatmap for five candidate genes in the buds of 2074A, 2074B, and R186 at the early abortion stage and abortion stage. **e** The heatmap for four candidate genes in the buds of 2074A, 2074B, E5903, R144, and R245 at the early abortion stage and abortion stage. R186_2, the R186 buds with diameters of 1.5–9.0 mm (the stage of pollen abortion). 2074A_2, the 2074A buds with diameters of 1.5–9.0 mm (the stage of pollen abortion). 2074B_2, the 2074B buds with diameters of 1.5–9.0 mm (the stage of pollen abortion)

In order to identify *Rf* candidate genes in the mapping interval, RNA-seq of 2074A, R186, E5903, R144 and R245 buds with diameters of 0–1.5 mm (the earlier stage of pollen abortion) and 1.5–9.0 mm (stage of pollen abortion) were performed. The total DEGs of R186_2 vs 2074A_2 and R186_2 vs 2074B_2 were 12,166 and 21,483 while the up-regulated DEGs in restorer lines were 8191 and 10,798 in R186_2 vs 2074A_2 and R186_2 vs 2074B_2, respectively (Fig. 4a-b). The VENN diagram revealed that the *GH_D05G3183*, *GH_D05G3265*, *GH_D05G3384*, *GH_D05G3388* and *GH_D05G3490* genes from the BSA interval had significantly higher expression levels in R186 than 2074A and 2074B (Fig. 4c). Figure 4d showed that the expression levels of the genes *GH_D05G3183*, *GH_D05G3265*, *GH_D05G3384* and *GH_D05G3490* were highest in the abortive stage buds of R186. The heatmap analysis found that the expression of *GH_D05G3183*, *GH_D05G3265*, *GH_D05G3384* and *GH_D05G3490* genes in buds with stage of pollen abortion of E5903, R144 and R245 were higher than 2074A and 2074B (Fig. 4e).

### Sequence, evolutionary and tissue differential expression analysis of candidate genes

Four candidate genes were screened by BSA and RNA-seq analysis, including *GH_D05G3183*, *GH_D05G3265*, *GH_D05G3384* and *GH_D05G3490*. Sequence analysis revealed that there are two SNPs in the exon of *GH_D05G3183*, including SNP_D05_35158622 and SNP_D05_35160174. Nucleotides of 2074A (sterile bulk) and R186 (fertile bulk) at SNP_D05_35158622 are T and A, respectively, which changes the amino acid from K to M (Table 4). Nucleotides of 2074A (sterile bulk) and R186 (fertile bulk) at SNP_D05_35160174 are G and C, respectively, which changes the amino acid from F to L (Table 4). Furthermore, there are two, seven and two SNPs that can cause changes in amino acids in exons of *GH_D05G3265*, *GH_D05G3384* and *GH_D05G3490* genes, respectively (Table 4). Nucleotides of 2074A and sterile bulk at those large effect variants (LEVs) are the same as the reference genome while R186 and fertile bulk are alternative nucleotides (Table 4). The index of all LEVs for *GH_D05G3183*, *GH_D05G3265*, *GH_D05G3384* and *GH_D05G3490* genes in fertile bulk are 1 while those in sterile bulk are 0 (Table 4).

**Table 4:** Haplotype analysis of non-synonymous variants in *Rf* candidate genes

Gene ID	Nucleotide Location	Ref	Alt	Amino acid position	Amino acid change	Index_Bulk_S	Index_Bulk_F	Δindex
*GH_D05G3183*	D05:35,158,622	T	A	336	K to M	0	1	1
*GH_D05G3183*	D05:35,160,174	G	C	14	F to L	0	1	1
*GH_D05G3265*	D05:38,391,931	G	A	162	H to Y	0	1	1
*GH_D05G3265*	D05:38,392,410	C	T	64	A to T	0	1	1
*GH_D05G3384*	D05:47,351,836	A	G	551	I to T	0	1	1
*GH_D05G3384*	D05:47,351,896	C	G	531	S to T	0	1	1
*GH_D05G3384*	D05:47,352,550	C	A	313	R to M	0	1	1
*GH_D05G3384*	D05:47,352,692	C	T	266	G to S	0	1	1
*GH_D05G3384*	D05:47,353,010	C	A	160	V to F	0	1	1
*GH_D05G3384*	D05:47,353,066	G	A	141	A to V	0	1	1
*GH_D05G3384*	D05:47,353,350	C	G	70	E to Q	0	1	1
*GH_D05G3490*	D05:51,181,181	A	T	51	M to L	0	1	1
*GH_D05G3490*	D05:51,182,087	G	C	111	V to L	0	1	1

Bulk_F, (2074A × R186) F_2_-fertile. Bulk_S, (2074A × R186) F_2_-sterile

**Fig. 5:**
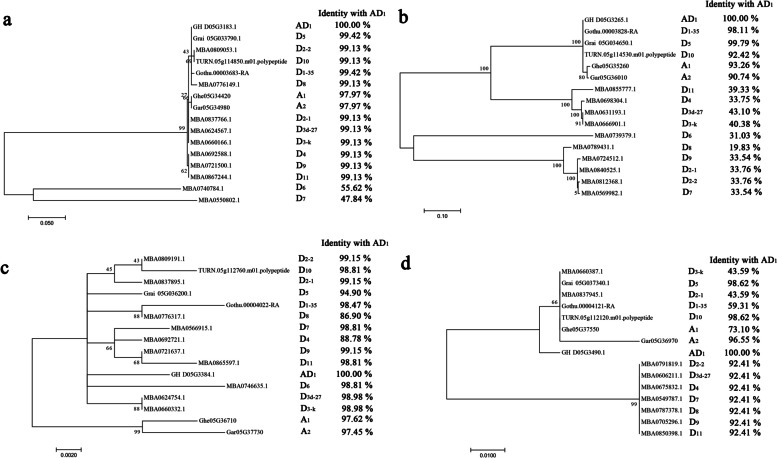
The evolutionary trees of 4 candidate proteins. Phylogenetic analyses of **a** GH_D05G3183, **b** GH_D05G3265, **c** GH_D05G3384, and **d** GH_D05G3490 proteins in *G. herbaceum* (A_1_), *G. arboreum* (A_2_), *G. hirsutum* (AD_1_) and all D genome cotton species

The phylogenetic analysis revealed that the evolutionary relationship between GH_D05G3183, GH_D05G3384 and their homologues from D_2-2_ are all relatively close while the evolutionary relationship between GH_D05G3265, GH_D05G3490 and their homologues from D_2-2_ are relatively distant (Fig. 5). The analysis of sequence identity found that the identity between GH_D05G3183, GH_D05G3384, GH_D05G3490 and their homologues from D_2-2_ are high while the identity between GH_D05G3265 and its homologue from D_2-2_ is only 33.76% (Fig. 5). *GH_D05G3183*, *GH_D05G3384* and *GH_D05G3490* genes were further identified as candidate genes for fertility restoration based on phylogenetic and sequence identity analysis.

**Fig. 6:**
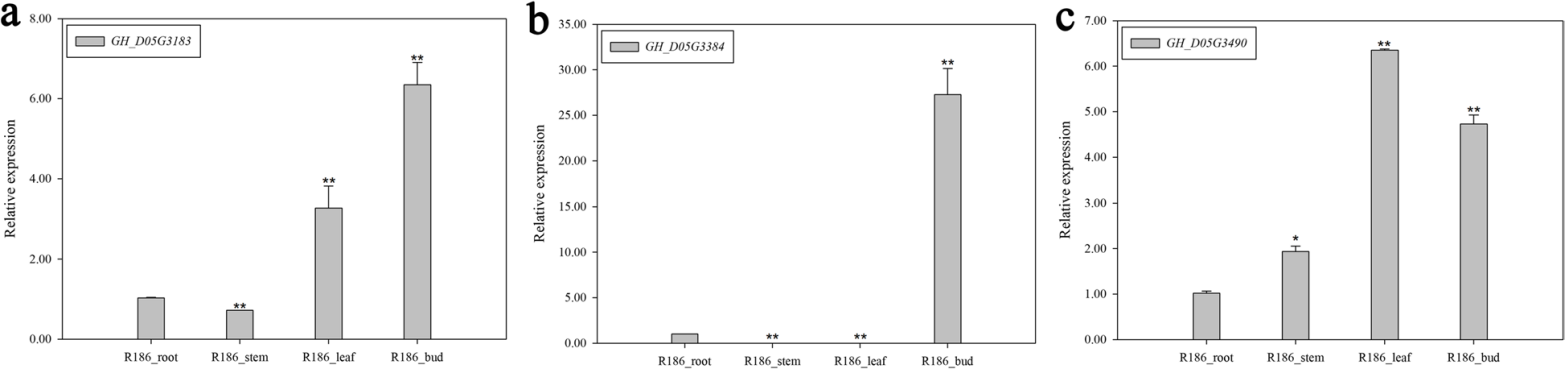
qRT-PCR analysis of *GH_D05G3183*, *GH_D05G3384* and *GH_D05G3490* in the roots, stems, leaves and buds of R186. **a** *GH_D05G3183*. **b** *GH_D05G3384*. **c*** GH_D05G3490*

The expression characteristics of the genes *GH_D05G3183*, *GH_D05G3384* and *GH_D05G3490* in multiple organs of the restorer line R186 were further analyzed by qRT-PCR. *GH_D05G3183* gene expression in buds was 6.17, 8.55 and 1.79 times higher than in root, stem and leaf, respectively (Fig. 6a). *GH_D05G3384* gene expression in bud was 27.57 times higher than in roots while *GH_D05G3384* gene expression was not detected in stem and leaf (Fig. 6b). Although *GH_D05G3490* gene expression was higher in leaf than in bud, its expression was higher in bud than in root and stem (Fig. 6c).

Eventually, *GH_D05G3183*, *GH_D05G3384,* and *GH_D05G3490* were identified as *Rf* candidate genes. The *GH_D05G3183* gene, which encodes purple acid phosphatase 3, was annotated with acid phosphatase activity. The *GH_D05G3384* gene encodes the putative protein NRT1/PTR FAMILY 2.14 with transmembrane transporter activity. DNA-directed RNA polymerases II, IV and V subunit 8B encoded by the *GH_D05G3490* gene has DNA-directed RNA polymerase activity (Table 5).

**Table 5:** Annotation information of CMS-Rf candidate genes

Gene ID	Gene Name	Gene Description	GO	Arabidopsis annoation
*GH_D05G3183*	PAP3	Purple acid phosphatase 3	Acid phosphatase activity	Purple acid phosphatase 3
*GH_D05G3384*	NPF2.14	Putative protein NRT1/ PTR FAMILY 2.14	Transmembrane transporter activity	Major facilitator superfamily protein
*GH_D05G3490*	NRPB8B	DNA-directed RNA polymerases II, IV and V subunit 8B	DNA-directed RNA polymerase activity	RNA polymerase Rpb8

## Discussion

### Elite CMS lines and restorer lines are the important parts of three-line breeding

As the male parent of the hybrid F_1_, the restoring power and agronomic traits of the restorer line have a significant impact on the hybrid F_1_. Screening of restorer lines with strong restoring power can contribute to the utilization of heterosis. R186 (Additional file 1, Supplemental Fig. 1b), a strong restorer line in this research, was selected by analyzing the fertility of 16 (2074A × R) F_1_ and possesses the strongest restoring power among 16 (2074A × R) F_1_ (Additional file 7, Supplemental Table 10). Although F_2_ derived from female parent upland cotton CMS line 3096 and male parent restorer line 866 had been used to locate fertility restorer genes by BSA [37], type of abortion cytoplasm of sterile lines between 3096 and 2074A are completely different. The CMS line (A) with CMS-D_8_ cytoplasm in Feng [38] and the CMS line (6001A) with *Gossypium thurberi* cytoplasm [26] are also different from 2074A in this research. In addition to the sterile line, another important difference is the restorer line. R186 is completely different from 866R [37], restorer line (R) [38] and the restorer line (7R13) [26].

### D_2-2_ CMS fertility restorer genes are located on chromosome D05

Many types of molecular markers, such as SNP [37, 38], InDel [39, 40], RAPD [32–34], SSR [32, 34], STS [34, 35], and AFLP [36] are effective tools for genetic mapping of fertility restoration genes. As sequencing cost has come down and technology has been upgraded, SNP and InDel have been the most popular molecular markers. In this research, the genetic location of *Rf* genes from R186 was determined by BSA, based on 805,401 SNPs and 80,717 InDels (Additional file 4, Supplemental Table 6), respectively. The long arm of chromosome 4 (A subgenome) was determined to be the genetic location of *Rf1* [32]. However, the *Rf* genes were considered to be located on chromosome D, since the abortive cytoplasm was derived from D_2-2_. Wang [30] delimited *Rf1* and *Rf2* on chromosome D05 with the help of four SSR markers. Zhao [37] found that the fertility of upland cotton CMS line 3096 was dominated by the locus of *Rf* genes located on 1.35 Mb of chrD05. The *Rf* genes dominating sterile line (A) with CMS-D_8_ cytoplasm were mapped on a 1.48 Mb interval of chromosome D05 [38]. The *Rf* genes for CMS line (6001A) derived from the crossing progenies of *G. thurberi* (D_1_) and AD_1_ were located in the interval of 2.05 Mb (53,632,812–55,682,586 bp) [26]. In this research, the *Rf* genes from R186 was delimited in a 4.30 Mb interval of chromosome D05. From our BSA results, it can be determined that the *Rf* genes for 2074A were mapped on chromosome D05, which was consistent with the location of the *Rf* genes for CMS lines with AD_1_, D_1_ and D_8_ abortive cytoplasm.

### The fertility of 2074A is controlled by multiple nuclear genes

The fertility of most reported cytoplasmic male sterile lines is controlled by single gene in plants [8, 24, 48–52,] or double [16, 18, 53–56]. However, the fertility of CMS-Charrua (C) maize and *Triticum timopheevii* (T)-type CMS wheat are dominated by multiple genes [25, 57]. 2074A, a cotton cytoplasmic male sterile line, possesses the nucleus of AD_1_ and the abortive cytoplasm of D_2-2_. Weaver [27] and Zhang [28] found that there is one restorer gene in D2R while Gao [26] believed that sterility of CMS-D_2-2_ cotton can be rescued by multiple genes. In this research, chi-square analysis results showed that multiple nuclear genes dominate the fertility of 2074A. The discovery is consistent with the views of Gao [26]. Zhang [58] revealed that orf610a can lead to excessive accumulation of reactive oxygen species, reduction in ATP content and inhibition of cellular growth of yeast and abnormal development of male reproductive organs in Arabidopsis. The qRT-PCR result revealed that the expression of *orf610a* in R186 buds with abortive cytoplasm was extremely significantly lower than that of 2074A, suggesting that the *Rf* genes may restore male fertility by reducing the expression level of *orf610a* in mitochondria (Additional file 2, Supplemental Fig. 2a). The cytoplasm of 2074B is normal, so the expression of *orf610a* in 2074B was almost undetectable (Additional file 2, Supplemental Fig. 2a). To investigate the potential mechanism of the three fertility restoration genes affecting male fertility, the interaction between the three fertility restoration genes and orf610a was analyzed by STRING V11.5 [59]. Prediction of protein interaction showed that GH_D05G3183, GH_D05G3384 and GH_D05G3490 may co-regulate *orf610a* in mitochondria by interacting with GH_A02G1295, thereby regulating male fertility of 2074A (Additional file 2, Supplemental Fig. 2b). We speculated that the genes *GH_D05G3183*, *GH_D05G3384* and *GH_D05G3490* co-regulated the fertility restoration of D_2-2_ abortion. Follow-up works will focus on simultaneous silencing of those genes to validate the relationship between *GH_D05G3183*, *GH_D05G3384* and *GH_D05G3490* genes and fertility restoration.

### The relationship between the PPR genes and 2074A fertility restoration

PPR genes, one of the largest gene families in land plants, function by targeting mitochondria or chloroplast, binding, editing, and processing organelle transcripts [60]. As *Rf* genes, PPR genes can restore the fertility of CMS-Boro II (BT) rice [53], CMS-Honglian (HL) rice [16, 55], CMS-wild-abortive (WA) rice [54], *Triticum timopheevii* (T)-type CMS wheat [57], Ogura (ogu) CMS oilseed rape [49, 50], Polima (pol) CMS oilseed rape [56], Kosena (kos) CMS radish [18], CMS-NJCMS1A soybean [52], Shahdara (Sha)-CMS Arabidopsis [51] and CMS-*pcf* petunia [48] at multiple different levels of regulation. In this research, only one PPR gene, *GH_D05G3465*, is localized in a 4.30 Mb interval on chromosome D05. However, the identity between GH_D05G3465 protein and its homologous proteins in D_2-2_ is low (Additional file 6, Supplemental Table 9). The expression of *GH_D05G3465* gene were not significantly different in 2074A, 2074B and R186 (Additional file 6, Supplemental Table 9). Although more than half of the reported Rf proteins belong to the PPR family, there are also many non-PPR Rf proteins in plants such as ACPS-like domain containing protein [23], glycine-rich protein [24], aldehyde dehydrogenase [21], bHLH transcription factor [25], transcription factors of the plant DREB1 family [8] and peptidase-like protein [20]. It can be determined that the types of CMS restorer genes are diverse. Based on the results of this research, valuable PPR fertility restoration candidate genes could not be discovered by BSA. *Rf* gene of 2074A may not be limited to PPR genes.

### The characteristics of CMS fertility restoration genes

The primary sequences of the proteins encoded by *Rf* genes are usually different between the sterile line and the restorer line [25]. Most *Rf* genes restore the fertility of CMS lines by positive regulation and usually have high expression levels in the restorer lines but low expression in the sterile lines [16, 18, 20, 21, 24, 25, 48–50, 53–57]. The expression level of the *Rf* genes in the restorer line should be significantly higher than that in the maintainer line, since the maintainer line does not contain the *Rf* genes. In this research, *GH_D05G3183*, *GH_D05G3265*, *GH_D05G3384* and *GH_D05G3490* genes with LEVs were most highly expressed in abortive buds of R186 and were screened as *Rf* candidate genes from 77 genes located in the 4.30 Mb interval of chromosome D05 with the assistance of R186, 2074B and 2074A bud transcriptome data (Fig. 4). Since the abortive cytoplasm of 2074A originated from D_2-2_, the *Rf* genes should exist in D_2-2_ nuclear genome. The evolutionary relationship or identity of Rf proteins from AD_1_ and D_2-2_ should be high. In the present study, *GH_D05G3183*, *GH_D05G3265*, *GH_D05G3384* and *GH_D05G3490* genes were identified as *Rf* candidate genes while *GH_D05G3265* gene was ruled out as *Rf* candidate gene, based on phylogenetic and sequence identity analysis (Fig. 5). Restorer genes are usually highly expressed in the stamens [8, 56]. In this work, *GH_D05G3183*, *GH_D05G3384* and *GH_D05G3490* genes were retained by qRT-PCR as a result of the high expression level of the 3 genes in buds (Fig. 6). The mechanism of *GH_D05G3183*, *GH_D05G3384* and *GH_D05G3490* genes affecting the male fertility of 2074A and the mining of other *Rf* genes that control 2074A male fertility based on the whole genome sequencing of R186 will be the focus of follow-up research.

## Conclusions

In the present study, genetic analysis revealed that male fertility in 2074A could be regulated by multiple *Rf* genes. The *Rf* loci were localized in a 4.3 Mb interval of chromosome D05. The genes *GH_D05G3183*, *GH_D05G3384* and *GH_D05G3490* were identified as *Rf* candidate genes based on RNA-seq, sequence and evolutionary analyses. Protein interaction analysis revealed that GH_D05G3183, GH_D05G3384 and GH_D05G3490 might restore male fertility in 2074A by co-regulating orf610a in mitochondria. Our study laid a foundation for exploring the *Rf* genes in D_2-2_ cytoplasmic male sterility and clarifying the mechanism of the *Rf* genes.

## Methods

### Plant materials and growth conditions

2074A, a cotton CMS line, possesses *G. harknessii* Brandegee CMS-D_2-2_ cytoplasm originated from DES-HAMS 277 and AD_1_ nucleus with no *Rf* genes (Additional file 1, Supplemental Fig. 1a) [10, 13]. R186 (Additional file 1, Supplemental Fig. 1b) was selected as strong restorer line through the male fertility identification of F_1_ produced by the hybrid of CMS-D_2-2_ line and restorer lines from 16 restorer lines (Additional file 7, Supplemental Table 10) in the summer of 2019 at Hejian Guoxin Cotton Base (Cangzhou City, China) (38°38′N, 116°13′E).

The F_2_ population derived from the 2074A and R186 hybrids, together with the BC_1_F_1_ population derived from the 2074A and R186 parents, was used to perform a genetic analysis of the restoring genes in cotton. (2074A × R186) F_2_ was planted in the winter of 2019 at Sanya Base of Cotton Research Institute of Chinese Academy of Agricultural Sciences (Sanya, China) (18°34′N, 109°65′E) and in the summer of 2020, 2021 at Hejian Guoxin Cotton Base. 2074A × (2074A × R186) was planted in the winter of 2019 at Sanya Base and in the summer of 2020 at Hejian Guoxin Cotton Base.

(2074A × R186) F_2:3_ was planted in the summer of 2020 at Hejian Guoxin Cotton Base. The F_2:3_ population, together with (2074A × R186) F_2_ planted in the winter of 2019 at Sanya Base, was used for fertility survey and BSA sampling. 2074A and R186 were planted in the summer of 2019 at Hejian Guoxin Cotton Base and used for RNA-seq sampling.

### Fertility investigation and plant sampling

The morphological standard of flower fertility is divided into three levels, such as fully fertile (full pollen), partially fertile (less pollen) and completely sterile (no pollen). There are also three types for fertility of plant individuals including fully fertile individuals (all flowers of individual are fertile), partially fertile individuals (individual possesses fertile and sterile flowers) and completely sterile individuals (all flowers of individual are sterile).

The fresh leaves of 19 extremely fertile individuals and 30 extremely sterile individuals from (2074A × R186) F_2_ population were collected for DNA extraction and BSA, based on the fertility survey results of (2074A × R186) F_2_ and (2074A × R186) F_2:3_ population. The fertility of the F_2:3_ lines from the extremely fertile F_2_ individuals must be fully fertile. The DNA of the extremely fertile and extremely sterile individuals were used to construct of the BSA extremely fertile and sterile bulks, respectively. Fresh leaves of 2074A and R186 were also used to extract DNA and were taken as the parents for BSA.

The buds of 2074A, R186, E5903 (restorer line), R144 (restorer line) and R245 (restorer line) with diameters of 0–1.5 mm (the earlier stage of pollen abortion) and 1.5–9 mm (stage of pollen abortion) [61] were collected in three biological replicates for RNA-seq.

### Whole genome sequencing and BSA

The DNA of cotton leaves including extremely fertile and extremely sterile individuals of (2074A × R186) F_2_, 2074A and R186 was isolated by cetyltrimethylammonium bromide (CTAB) method [62]. When the qualified sample DNA was prepared, the library was constructed in strict accordance with the protocol provided by the kit of NEBNext® Ultra™ II DNA Library Prep Kit for Illumina® (NEB). Sequencing could be performed on the Illumina HiSeq platform when the library quality met the requirements. The library quality was tested as follows: First, Qubit3.0 was used for preliminary quantification. Then, the insert size of the library was detected using Agilent 2100. The next experiment could only be performed after the insert size met the expectations and no connector contamination was present. Last, a qualified library whose effective concentration was more than 2 nM was obtained by accurately quantifying the effective concentration of the library using the German ANALYTIKJENA (Jena) QTOWER real-time fluorescent quantitative PCR instrument (German). The library was pooled, and paired-end 150 bp (PE150) sequencing was performed on the Illumina HiSeq platform. The raw data obtained by sequencing was transformed into clean data by a three-step filter. (i) Linker sequence contained in reads was removed using ‘cutadapt’ software (1.13); (ii) Low-quality bases in reads were eliminated by ‘trimmomatic’ software (0.36); (iii) The length of reads must be greater than 50 bp. The MEM algorithm of ‘BWA’ software (0.7.15-r1140) was used to align clean reads to the reference AD_1_ genome [43] and the result file was output in SAM format. The SAM format file was converted to BAM format with ‘samtools’ software (1.3.1). The final BAM file could be used for statistics of coverage and depth and variant calling after reads in the BAM file being sorted by SortSam of Picard tool (1.91). The HaplotypeCaller module in the GATK (3.7) software package was used to generate gvcf files for each sample, and then variants detection (SNPs and InDels) of all samples were performed using the GenotypeGVCFs module. The variation information output by GATK was stored in a file in vcf format, which contains all the variations present between the sample and the reference AD_1_ genome. In order to analyze the variants between samples, the original mutations were screened based on the following criterion: (i) The sequencing depth of the parent is not less than 5; (ii) The sequencing depth of the bulks is not less than 10; (iii) The parents are all homozygous and there are polymorphisms among the parents; (iv) The SNP-index value of bulks cannot be more than 0.8 or less than 0.2 at the same time. ANNOVAR software (2016Feb1) [63] was used to annotate variants and predict the effect of variants on gene function. There are two algorithms suitable for the location of *Rf* locus in BSA, including Δ(SNP-index) and ED. The ED algorithm, also called MMAPPR, calculates the frequency distance of each mutant between different bulks, and uses the distance difference to reflect the linkage strength between marker and target interval [64]. DeepBSA software was used to calculate the Δ(SNP-index) of each mutation site and evaluate ED between mutation sites based on default parameters [65]. The LEVs in the candidate interval were focused on. LEVs are mutations that cause changes in the protein sequence, including non-synonymous SNP, frameshift InDel, non-frameshift InDel, stop-gain SNP/InDel stop-loss SNP/InDel, and splicing.

### RNA-seq analysis

The total RNA was extracted from buds by CTAB-ammonium acetate method with slight modifications [66]. The detection of RNA samples mainly includes four methods: (i) The contamination and degradation of RNA were monitored on 1% agarose gels; (ii) RNA purity (OD260/280) was checked by the NanoPhotometer® spectrophotometer (IMPLEN, CA, USA); (iii) The concentration of RNA was determined by Qubit® RNA Assay Kit in Qubit® 2.0 Flurometer (Life Technologies, CA, USA); (iv) The RNA Nano 6000 Assay Kit of the Bioanalyzer 2100 system (Agilent Technologies, CA, USA) was used to assess RNA integrity. A total amount of 3 µg RNA each sample was prepared as input material for the RNA-seq. Sequencing libraries were constructed with the assistance of NEBNext® Ultra™ RNA Library Prep Kit for Illumina® (NEB, USA) following manufacturer’s recommendations. After the library was constructed, Qubit2.0 was used for preliminary quantification, and the library was diluted to 1 ng·ul^−1^. The inserting size of library was then evaluated using the Agilent Bioanalyzer 2100 system. The Q-PCR method was used to accurately quantify the effective concentration of the library (the effective concentration of the library > 2 nM) to ensure the quality of the library after insert size of library meeting expectations. Sequencing was performed on an Illumina Hiseq platform when different libraries were pooled according to the requirements of library effective concentration and the target off-machine data volume. Raw data obtained from HiSeq sequencing was transformed into clean data by a three-step data processing. (i) Removing reads with adapters; (ii) Removing reads with more than 10% uncertain bases; (iii) Removing low-quality reads. At the same time, the Q20, Q30, and GC content of the clean data were calculated. High-quality clean data was the basis for all the downstream analyses. STAR was used to align paired-end clean reads to the reference cotton genome [43]. The length of the gene and reads count mapped to this gene were the foundation of calculating expected number of Fragments Per Kilobase of transcript sequence per Millions base pairs sequenced (FPKM) of per gene [67]. DESeq R package (1.18.0) was used to perform differential expression analysis of genes, and the threshold for significantly differential expression of genes is *P*-value of 0.05 and |log_2_(Fold change)|≥ 1.

### Application of public transcriptome data

RNA-seq data of the maintainer line 2074B were referred to Nie [68].

### Functional enrichment analysis

Functional enrichment analysis of candidate genes was performed at online website GO (http://www.geneontology.org/).

### qRT-PCR analysis

RNA from root, stem, leaves and the buds with diameters of 1.5–9 mm of R186 was extracted by the CTAB method mentioned above [66], which was used for expression verification of candidate genes.

PrimeScript™ RT reagent Kit with gDNA Eraser (Perfect Real Time) was used to complete the reverse transcription of RNA. The experiment of qRT-PCR was executed with the assistance of PrimeScript™ RT reagent Kit (Perfect Real Time). The primers used in the experiments are shown in Additional file 8, and Supplemental Table 11. Three replicates were set for each sample and *GhUBQ7* (GenBank accession number: DQ116441) was the internal reference gene in all qRT-PCR experiments. 2^−ΔΔCt^ method was adopted to calculate the relative expression level of each gene [69].

### Homology, evolutionary and protein interaction analysis

The homologous proteins sequence of the candidate proteins in A_1_, A_2_, AD_1_ and all D genome cotton were searched and downloaded from the CottonGen (https://www.cottongen.org) [70]. The identity between homologous proteins of different cotton species was calculated using the DNAMAN software. Evolutionary analysis between homologous proteins was performed with the assistance of the MEGA 7.0.26 software.

Protein interaction analysis of candidate proteins with reported mitochondrial abortive orf610a in cotton [58] were predicted based on their homologous proteins in *Gossypium raimondii* L. using STRING V11.5 [59]. The minimum required interaction score was set to 0.150.

### Statement

Complying *with the IUCN Policy Statement on Research Involving Species at Risk of Extinction* and *the Convention on the Trade in Endangered Species of Wild Fauna and Flora*, we confirm that the plant materials used in the present study does not involve any species at risk of extinction. All methods performed are in accordance with the relevant institutional, national, and international guidelines and legislation.

The cytoplasmic male sterile lines DES-HAMS277, DES-HAMS16 and the restorer lines DES-HAF277 and DES-HAF16 are originated from *Gossypium harknessii* and were released since 1970s [10]. All these lines were introduced into China in 1980 by Dr. Tianjue Zuo, and the seeds were divided into two parts. One was sent to the Institute of Cash Crops, Hubei Academy of Agricultural Sciences, Wuhan 430,064, Hubei, China, and the other to the Institute of Cotton Research, Chinese Academy of Agricultural Sciences, Anyang 455,000, Henan, China. Using these original allo-cytoplasm lines serial new lines such as 2074A, R186 were developed in our lab and issued to China Agricultural University since 2005 [13, 14, 68, 71]. We confirm that all the introduced processes have been authorized.

## Supplementary Information

**Additional file 1.** Supplemental figure 1

**Additional file 2.** Supplemental figure 2

**Additional file 3.** Supplemental table 1-4

**Additional file 4.** Supplemental table 5-6

**Additional file 5.** Supplemental table 7-8

**Additional file 6.** Supplemental table 9

**Additional file 7.** Supplemental table 10

**Additional file 8.** Supplemental table 11

## Data Availability

Sequencing data have been uploaded to GSA of the NGDC website (https://ngdc.cncb.ac.cn/sso/login?service=https://ngdc.cncb.ac.cn/gsa/login). The accession numbers of GSA are CRA007637 and CRA007640, respectively: CRA007637 is for BSA data (https://ngdc.cncb.ac.cn/gsa/s/E7jGRJ7p), and CRA007640 is for RNA-seq data (https://ngdc.cncb.ac.cn/gsa/s/64zE2c3u). R186, 2074A, (2074A × R186) F_2_ in the manuscript are accordingly recorded using their line numbers: 19A1114, 19A1117, 19A1102, respectively.

## Abbreviations

CMS: Cytoplasmic male sterility
BSA: Bulked segregant analysis
GMS: Genic male sterility
ROS: Reactive oxygen species
Rf: Restorer of fertility
PPR: Pentatricopeptide repeat
ED: Euclidean distance
LEVs: Large effect variants
FPKM: Expected number of Fragments Per Kilobase of transcript sequence per Millions base pairs
GO: Gene ontology
qRT-PCR: Real-time quantitative PCR
