# Supplementary figures and images for: Identification of fertility restoration candidate genes from a restorer line R186 for Gossypium harknessii cytoplasmic male sterile cotton
Source: BMC Plant Biol. 2023 Apr 4;23:175. doi: 10.1186/s12870-023-04185-z (PMC10071737; doi:10.1186/s12870-023-04185-z)

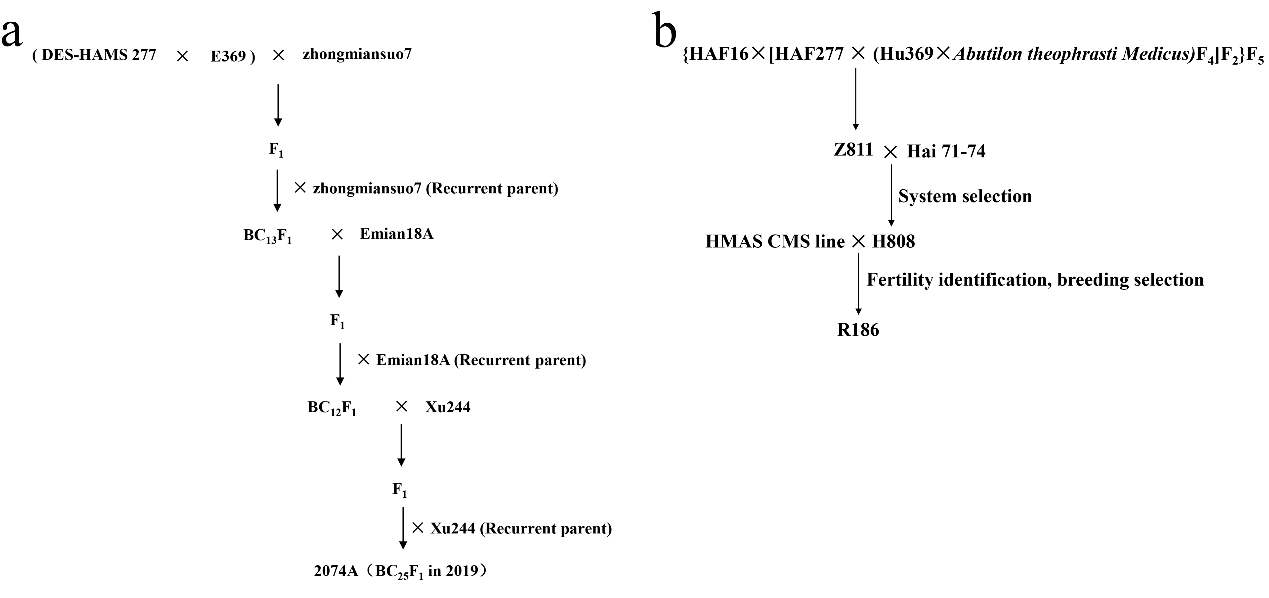


**Supplemental Figure 1.** The pedigree of 2074A and R186. **a** 2074A. **b** R186

Supplement: Supplementary file 1 — Additional file 1. Supplemental figure 1 [file 12870_2023_4185_MOESM1_ESM.docx]
