# Supplementary material for: Identification of fertility restoration candidate genes from a restorer line R186 for Gossypium harknessii cytoplasmic male sterile cotton
Source: BMC Plant Biol. 2023 Apr 4;23:175. doi: 10.1186/s12870-023-04185-z (PMC10071737; doi:10.1186/s12870-023-04185-z)
Supplement: Supplementary file 2 — Additional file 2. Supplemental figure 2 [file 12870_2023_4185_MOESM2_ESM.docx]

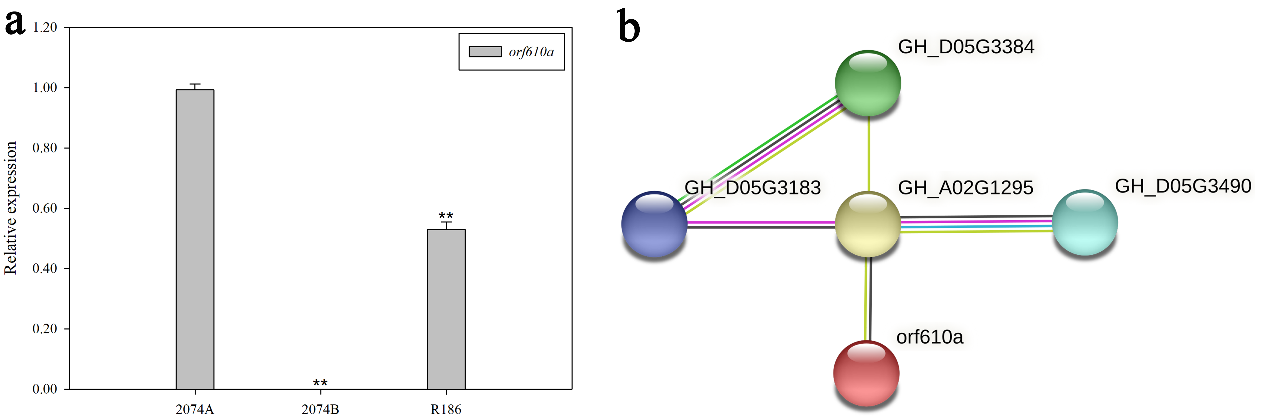


**Supplemental Figure 2.** GH_D05G3183, GH_D05G3384 and GH_D05G3490 regulate male fertility by affecting orf610a. **a** Analysis of *orf610a* gene expression level in 2074A, 2074B and R186 buds with a diameter of 1.5-9.0 mm by qRT-PCR. **b** Prediction of protein interaction between three key CMS-Rf candidate genes and *orf610a*. The different colored lines between the circles indicate the interactions of different sources of information, i.e., from curated databases (blue), experimentally determined (purple), co-expression (black), gene neighborhood (green), and by text mining (yellow)
